# Efficacy and safety of endoscopic submucosal tunnel dissection for superficial esophageal neoplastic lesions: a systematic review and meta-analysis

**DOI:** 10.1186/s13019-020-1074-9

**Published:** 2020-02-04

**Authors:** Wei Peng, Shali Tan, Yutang Ren, Huan Li, Yan Peng, Xiangsheng Fu, Xiaowei Tang

**Affiliations:** 1 Department of Gastroenterology, the Affliated Hospital of Southwest Medical University, Street Taiping No.25, Region Jiangyang, Luzhou, 646099 Sichuan Province China; 2 Departmemt of Gastroenterology, Beijing Tsinghua Changgung Hospital Medical Center, Tsinghua University, Beijing, China; 3 Department of Gastroenterology, the Affiliated Hospital of North Sichuan Medical College, Road Wenhua 63#, Region Shunqing, Nanchong, 637000 Sichuan China

**Keywords:** Endoscopic submucosal tunnel dissection, Esophageal neoplastic lesions, Systematic review, Meta-analysis

## Abstract

**Background:**

Presently, endoscopic submucosal tunnel dissection (ESTD) has been a novel therapy for superficial esophageal neoplastic lesions (SENL), especially for circumferential neoplastic lesions. A number of studies have reported the clinical application of ESTD with promising outcomes. Therefore, we conducted a systematic review and meta-analysis to evaluated the efficacy and safety of ESTD for SENL .

**Methods:**

From 2013 to November 2018, Pubmed, Embase and Cochrane databases were searched to determine studies reporting ESTD treatment of SENL. Weighted pooled rates (WPR) were calculated for en bloc resection, R0 resection and complication of ESTD. Risk ratios (RR) were calculated and pooled to compare the clinical outcomes of ESTD with ESD for SENL.

**Results:**

A total of 9 studies involving 494 patients with 518 esophageal neoplastic lesions were included in our study. WPR for en bloc resection and R0 resection of ESTD was 97.0% (95% CI: 94.7–98.3%) and 84.1% (95% CI: 80.5–87.1%), respectively. WPR for complication was 40.0% (95% CI: 25.8–56.1%). Two studies with 265 patients compared the performance of ESTD with ESD. Pooled RR for en bloc resection and R0 resection was 1.04 (95% CI: 0.95–1.14, *P* = 0.42) and 1.01 (95% CI: 0.93–1.10, *P* = 0.73), respectively. Pooled RR for complication was 0.68 (95% CI: 0.46–1.01, *P* = 0.05).

**Conclusion:**

Our study showed that ESTD is effective for treating SENL with high en bloc resection rate and R0 resection rate, but accompanying by a relatively high complications.

## Background

Minimally invasive endoscopic techniques for the treatment of early esophageal neoplastic lesions currently include endoscopic mucosal resection (EMR), endoscopic piecemeal mucosal resection, and endoscopic submucosal dissection (ESD) [1–3]. Although EMR has become widely used as a conventional endoscopic method, some studies have reported that this option is associated with a high risk of local recurrence [4, 5]. As compared to EMR, ESD is a better choice for en bloc resection of superficial esophageal lesions and provides an accurate pathologic diagnosis [6, 7]. However, due to the relatively thin wall and narrow lumen of the esophagus, ESD poses a risk of some serious complications, especially perforation [7, 8]. According to the findings of a recent meta-analysis, the pooled perforation rate during esophageal ESD is 5.0% [9].

Inspired by the reported success of the submucosal tunnel endoscopic method, endoscopic submucosal tunnel dissection (ESTD) was developed as an alternative technique for the treatment of esophageal neoplastic lesions, especially circumferential superficial esophageal neoplastic lesions (SENLs) [10]. A number of single-center studies have reported promising outcomes with the clinical application of ESTD [11, 12]. Therefore, the primary aim of this systematic review and meta-analysis was to investigate the efficacy and safety of ESTD for SENLs in terms of the R0 resection rate, the en bloc resection rate, complications, and other parameters. In addition, the clinical outcomes between ESTD and ESD for the treatment of SENLs were compared.

## Methods

### Search strategy

This study was conducted in accordance with the guidelines of the Preferred Reporting Items for Systematic Reviews and Meta-Analyses [13]. A comprehensive literature search was conducted of the PubMed (https://www.ncbi.nlm.nih.gov/pubmed/), Embase (https://www.embase.com/), and Cochrane Library (https://www.cochranelibrary.com/) databases in November 2018 with the following keywords: “endoscopic submucosal tunnel dissection,” “esophageal neoplastic lesions,” “esophageal tumor,” “endoscopic submucosal dissection,” “endoscopic resection,” and “submucosal tunnel endoscopic resection.” When appropriate, Boolean operators (NOT, AND, OR) were used to widen or narrow the search range. The reference lists of the included studies were manually searched for relevant articles.

### Study selection

Two reviewers (W. Peng and S. Tan) independently screened all titles, abstracts, and full text of the retrieved articles for relevance to the study. The selected studies met the following inclusion criteria: (1) the subjects were patients with SENLs and treated with ESTD; (2) the R0 resection rate, the en bloc resection rate, complications, and follow-up duration were reported; and (3) full-text articles were available. The following types of articles were excluded from analysis: animal studies, case reports (number of subjects < 3), commentaries, general reviews, and conference abstracts.

### Data extraction

The following data were extracted from each study using a standardized data extraction sheet: (1) baseline characteristics, including the name of the first author, year of publication, country of origin, number of subjects, average age of subjects, male:female ratio, and study design; (2) clinical characteristics, including lesion location, Paris classification, number of lesions, average size of lesions, mean duration of the procedure, origin of the lesion, and the average follow-up duration; and (3) therapeutic outcomes, including the R0 resection rate, the en bloc resection rate, histology of the lesions, complications, and recurrence rate.

### Definitions

The following definitions were used in this study:

1. Paris classification: a standard typing method for morphological classification of early digestive tract cancers [14].
2. Complication: an adverse event due to ESTD for esophageal neoplastic lesions, such as esophageal stricture (ES) and muscular injury.
3. R0 resection: complete encapsulation of the lesion and the presence of tumor cells at the basal and lateral margins. In addition, no residual cancer tissue was found by endoscopic examination or biopsy during the follow-up period.
4. En bloc resection: the lesion was excised endoscopically and a single specimen was obtained.

### Assessment of study quality

The methodological quality of the included studies was independently assessed by two investigators (S. Huang and H. Li). The two investigators also applied the Newcastle–Ottawa Scale (NOS) for cohort studies to evaluate the methodological quality [15, 16]. The NOS uses a point system with a maximum of 9 points to evaluate three domains of a study: selection, comparability, and outcome. When there was a disagreement between the reviewers, a consensus was reached by discussion with a third reviewer (X. Fu).

### Statistical analysis

The en bloc and R0 resection rates were used to assess efficacy, and the complication rate was used to determine safety. The weighted pooled rate (WPR) of the primary outcomes of interest along with the 95% confidence interval (CI) were calculated. The I^2^ statistic and Cochran Q test were used to assess heterogeneity, where a *p* value < 0.1 for the Cochran Q test indicated the presence of heterogeneity. An I^2^ value of > 50% was considered to indicate significant heterogeneity. Publication bias was assessed via visual inspection of a funnel plot and the Egger’s test. Furthermore, the efficacy and safety of ESTD vs. ESD for the management of SENLs were compared. Risk ratios (RR) for en bloc resection, R0 resection, and complications were pooled using a random effects model. All statistical analyses were conducted using Comprehensive Meta-analysis software (version 3.0; Biostat, Englewood, NJ, USA).

## Results

### Search results

**Fig. 1:**
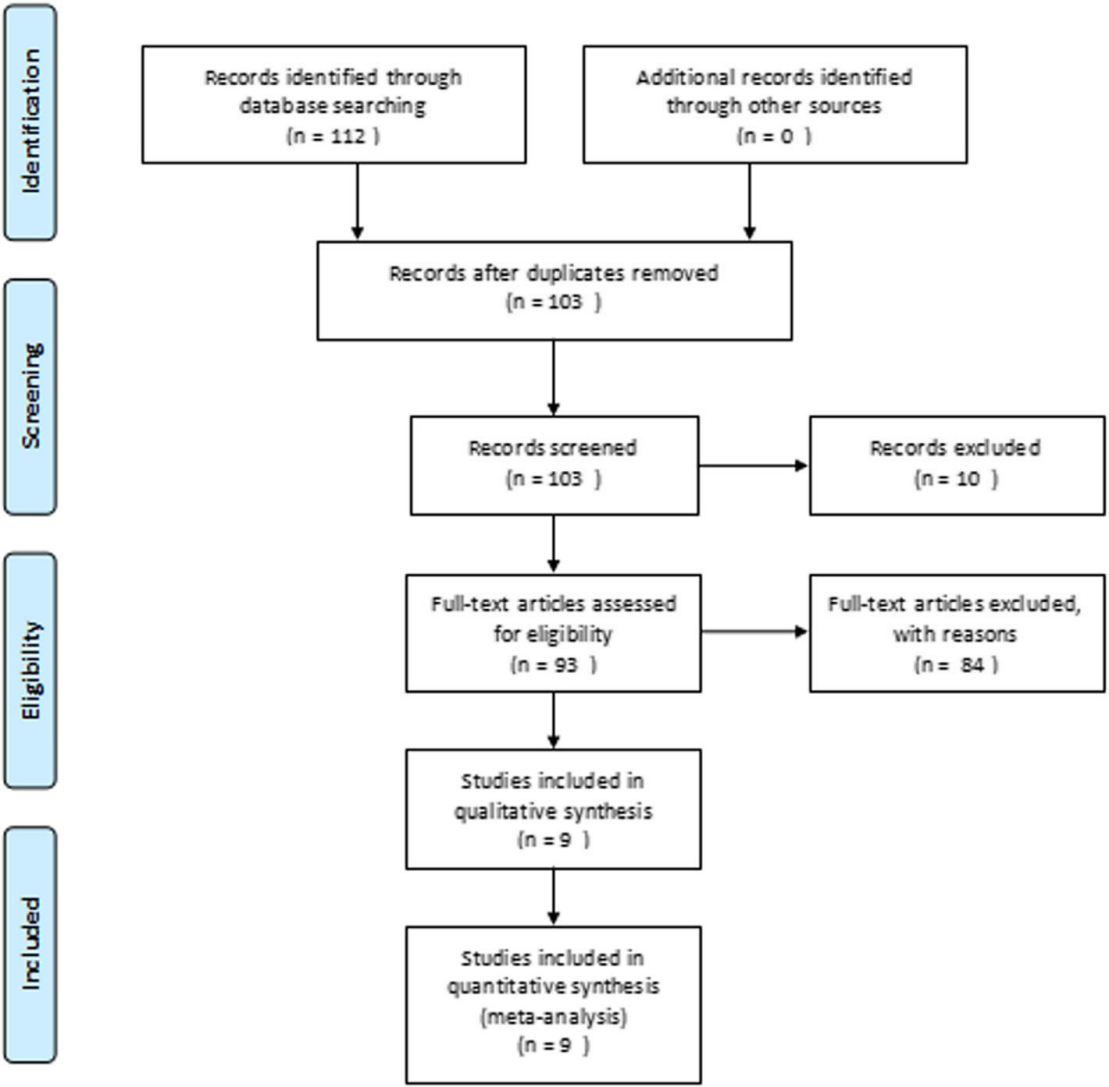
PRISMA flowchart for search strategy and selection of eligible studies

The database search yielded a total of 112 potential articles, while no further article was identified by a manual search of the reference lists. Finally, nine original articles met the inclusion criteria (Fig. 1). Of these included studies, three were prospective and six were retrospective [12, 17–24].

### Descriptive analysis

The baseline characteristics of the included studies are shown in Table 1. These studies were published from 2013 to 2018 and most were conducted in China. The studies included a total of 494 patients with 518 lesions. The clinical characteristics of the included studies are shown in Table 2. Regarding the lesion location, 35 (6.8%) lesions were located in the upper thorax, 1 (0.2%) in the upper to mid thorax, 279 (53.9%) in the mid thorax, 12 (2.3%) in the mid to lower thorax, and 166 (32.0%) in the lower thorax. According to the Paris classification to distinguish the macroscopic type of esophageal neoplastic lesions, 18 (3.5%) lesions were classified as type I, 139 (26.8%) as type IIa, 132 (25.5%) as type IIb, 43 (8.3%) as type IIc, 13 (2.5%) as type IIa + IIb, 54 (10.4%) as type IIa + IIc, 1 (0.2%) as type IIb + IIc, and 3 (0.6%) as type III (five studies did not mention the macroscopic type of the lesion). The mean length of the lesions was 39.1 mm. The average surgical duration was 97.0 min (the median surgical duration was not included in the calculation). The average follow-up duration was 19.8 months (four studies did not mention the follow-up duration, thus, the median follow-up was not included in the calculation).

**Table 1:** Baseline characteristics of the included studies

Authors	Year	Country	Subjects, n	Mean age, years	Males, n (%)	Type
Wang et al. [17]	2018	China	289	61.4	213 (73.7)	Retrospectively
Zhang et al. [18]	2018	China	46	62.3	29 (63.0)	Retrospectively
Zhang et al. [12]	2018	China	52	61.7	33 (63.5)	Retrospectively
Huang et al. [19]	2017	China	38	58.7	33 (86.8)	Retrospectively
Gan et al. [20]	2016	China	7	64.8	6 (85.7)	Prospectively
Ye et al. [21]	2016	China	23	62.3	16 (69.6)	Prospectively
Pioche et al. [22]	2013	France	11	64.8	9 (81.8)	Retrospectively
Linghu et al. [23]	2013	China	5	68.0	3 (60.0)	Retrospectively
Arantes et al. [24]	2013	Brazil	23	68.0	19 (82.6)	Prospectively

**Table 2:** Clinical characteristics of the included studies

Authors	Lesions, n	Location of the lesion					Paris classification								Mean size (mm)	Operation time (min)	Mean follow-up (mo)
		Ht	Ht + Mt	Mt	Mt + Lt	Lt	I	IIa	IIb	IIc	IIa + IIb	IIa + IIc	IIb + IIc	III			
Wang et al. [17]	311	24	0	200	0	87	18	111	94	35	0	50	0	3	Circumferential extent, n (1)	102.4	20.2
Zhang et al. [18]	46	3	1	16	6	20	0	19	18	2	3	4	0	0	Circumferential extent, n (2)	74.5	NA
Zhang et al. [12]	52	5	0	20	0	27	NA								Circumferential extent, n (3)	93.2	NA
Huang et al. [19]	38	3	0	22	0	13	NA								39.0 (M)	38.0 (M)	NA
Gan et al. [20]	7	0	0	3	4	0	0	0	1	0	5	0	1	0	61.4	121.1	7.3
Ye et al. [21]	23	0	0	10	0	13	0	8	9	6	0	0	0	0	65.0 (M)	145.0 (M)	16 (M)
Pioche et al. [22]	11	0	0	5	0	6	0	1	7	0	3	0	0	0	49.0	76.7	NA
Linghu et al. [23]	5	0	0	3	2	0	0	0	3	0	2	0	0	0	57.0	77.0	7.4
Arantes et al. [24]	25	NA					NA								25.0	85.0	21.4

*Ht* higher thoracic, *Mt* middle thoracic, *Lt* lower thoracic, *M* Median

Circumferential extent, n (1): ≤ 1/4 (3.5%); ≤ 1/2 (52.4%); ≤ 3/4 (20.9%); ≤ 7/8 (13.2%); ≤1 (10.0%)

Circumferential extent, n (2): ≥ 1/3, < 1/2 7 (15.2%); ≥ 1/2, < 3/4 17 (37.0%); ≥ 3/4, < 4/4 4 (8.7%); 4 /4 18 (39.1%)

Circumferential extent, n (3): ≥ 1/3, < 3/4 29 (55.77%); ≥ 3/4 23 (44.23%)

### Therapeutic outcome and complications

**Fig. 2:**
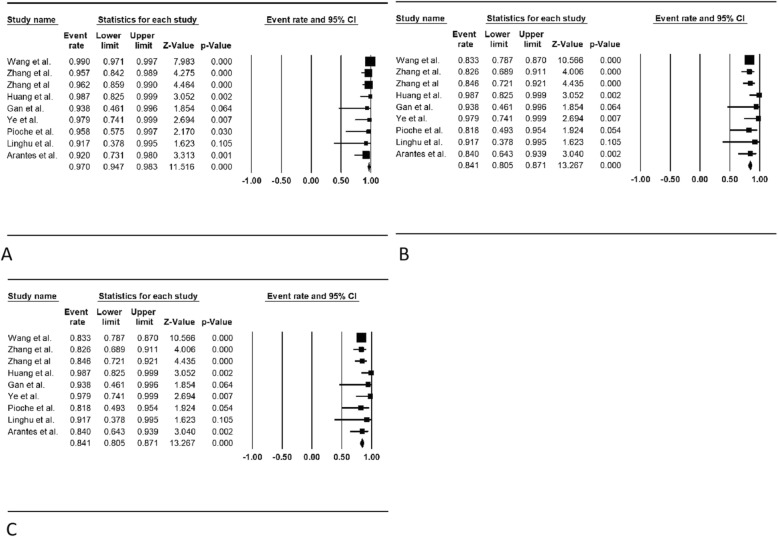
Forest plot for en bloc resection rate, R0 resection rate and complication rate of **endoscopic submucosal tunnel dissection (**ESTD). **a**, Weighted pooled rates (WPR) for en bloc resection and R0 resection of ESTD was 97.0% (95% CI: 94.7–98.3%).; **b**, Forest plot for R0 resection rate of ESTD was 84.1% (95% CI: 80.5–87.1%); **c**, WPR for complication of ESTD was 40.0% (95% CI: 25.8–56.1%)

**Fig. 3:**
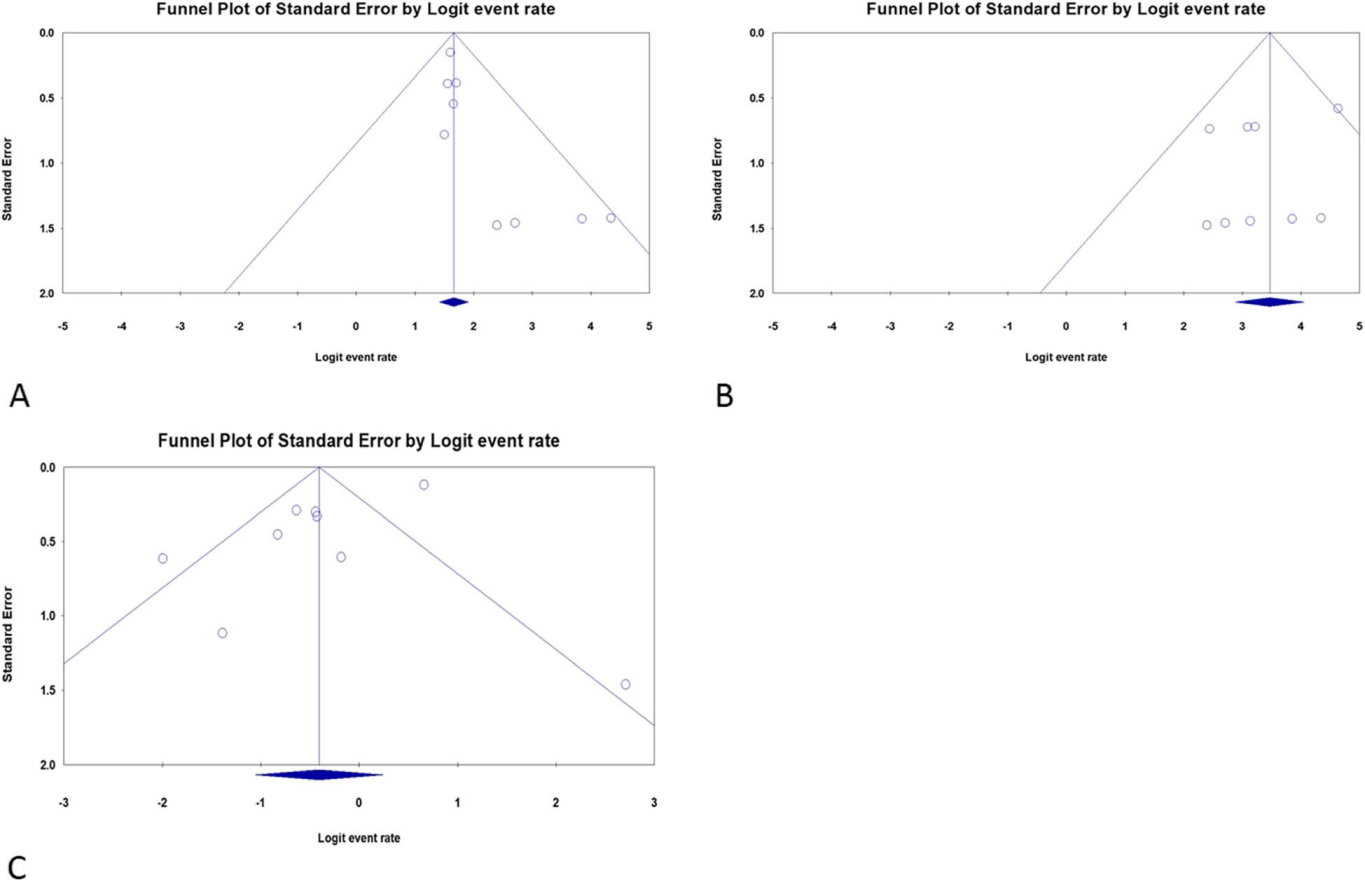
Funnel Plot for en bloc resection rate, R0 resection rate and complication rate of **endoscopic submucosal tunnel dissection**. **a**, We found publication bias for this estimate based on asymmetric funnel plot; **b**, No publication bias was detected based on asymmetric funnel plot; **c**. No publication bias was detected based on asymmetric funnel plot

The therapeutic outcomes of the included studies are shown in Table 3. The pooled WPR (95% CI) for en bloc resection with ESTD was 97.0% (94.7–98.3%, Cochran Q test *p* = 0.467, I^2^ = 0%, Fig.2a). Based on the asymmetry of the funnel plot and Egger’s test result (*p* = 0.02), there was publication bias for this estimate (Fig. 3 a). The pooled WPR (95% CI) for R0 resection with ESTD was 84.1% (80.5–87.1%; Cochran Q test *p* = 0.546, I^2^ = 0%, Fig. 2b). Based on the asymmetry of the funnel plot and Egger’s test result (*p* = 0.47), there was no publication bias (Fig. 3b). Complications occurred in 279 patients. The pooled WPR (95% CI) for complications was 40.0% (25.8–56.1%; Cochran Q test *p* < 0.05, I^2^ = 85.2%, Fig. 2c). Based on the asymmetry of the funnel plot and Egger’s test (*p* = 0.06), there was no publication bias (Fig. 3c). Complications of the included studies are shown in Table 4. The most common complications reported in the studies included muscular injury (30.5, 95% CI = 26–35.5%, I^2^ = 82.4%), esophageal stenosis (ES) (18.8 95% CI = 15.2–22.9%, I^2^ = 66.5%), and postoperative infection (10.4, 95% CI = 7.4–14.5%, I^2^ = 0%). The following complications and rates were reported in the included studies: perforation: 2.2% (95% CI = 1.2–4.3%), I^2^ = 0%; bleeding: 8.7% (95% CI = 5.9–12.5%), I^2^ = 0%; cardiac mucosal laceration: 6.1% (95% CI = 2.8–1.3%), I^2^ = 0%; chest pain: 10.5% (95% CI = 4.0–24.9%), I^2^ = 0%; pneumothorax: 4.3% (95% CI = 0.6–25.2%), I^2^ = 0%; and emphysema: 8.8% (95% CI = 3.7–19.4%), I^2^ = 0%. During the mean follow-up duration of 19.8 months, the tumor recurrence rate was 0.6% and one patient died due to cerebral infarction.

**Table 3:** Clinical outcomes of the included studies

Authors	En bloc resection, n (%)	R0 resection, n (%)	Histology, n (%)				Local Recurrences, n (%)	NOS
			LGIN	HGIN	SCC	Adca		
Wang et al. [17]	308 (99.0)	259 (81.3)	67	159	85	0	0	5
Zhang et al. [18]	44 (95.7)	38 (82.6)	2	14	30	0	0	6
Zhang et al. [12]	50 (96.2)	44 (84.6)	3	17	32	0	0	7
Huang et al. [19]	38 (100)	38 (100)	0	18	20	0	0	8
Gan et al. [20]	7 (100)	7 (100)	0	1	6	0	0	5
Ye et al. [21]	23 (100)	23 (100)	0	10	13	0	0	4
Pioche et al. [22]	11(100)	9 (81.8)	0	0	9	2	1 (9.1)	4
Linghu et al. [23]	5 (100)	5 (100)	0	3	2	0	0	4
Arantes et al. [24]	23 (92)	21 (84)	1	6	15	3	2 (8.7)	6

*LGIN* low-grade intraepithelial neoplasia;

*HGIN* how-grade intraepithelial neoplasia;

*SCC* squamous cell carcinoma;

*Adca*, adenocarcinoma;

*NOS* Newcastle-Ottawa Scale

**Table 4:** Rates of adverse outcomes in patients underwent endoscopic submucosal tunnel dissection

Adverse outcomes	Patients, n	Rate (95%CI), %	I^2^, %
Perioperative			
Perforation	9	2.2 (1.2, 4.3)	0
Bleeding	25	8.7 (5.9, 12.5)	0
Muscular injury	114	30.5 (26, 35.5)	82.4
Cardiac mucosal laceration	6	6.1 (2.8, 1.3)	0
Pneumothorax	1	4.3 (0.6, 25.2)	0
Emphysema	5	8.8 (3.7, 19.4)	0
Postoperative			
ES	85	18.8 (15.2, 22.9)	66.5
Infection	30	10.4 (7.4, 14.5)	0
Chest pain	4	10.5 (4.0, 24.9)	0

*ES* esophageal stricture;

*I*^*2*^ indicates percentage of heterogeneity of outcome estimates between included studies

### Meta-analysis

**Fig. 4:**
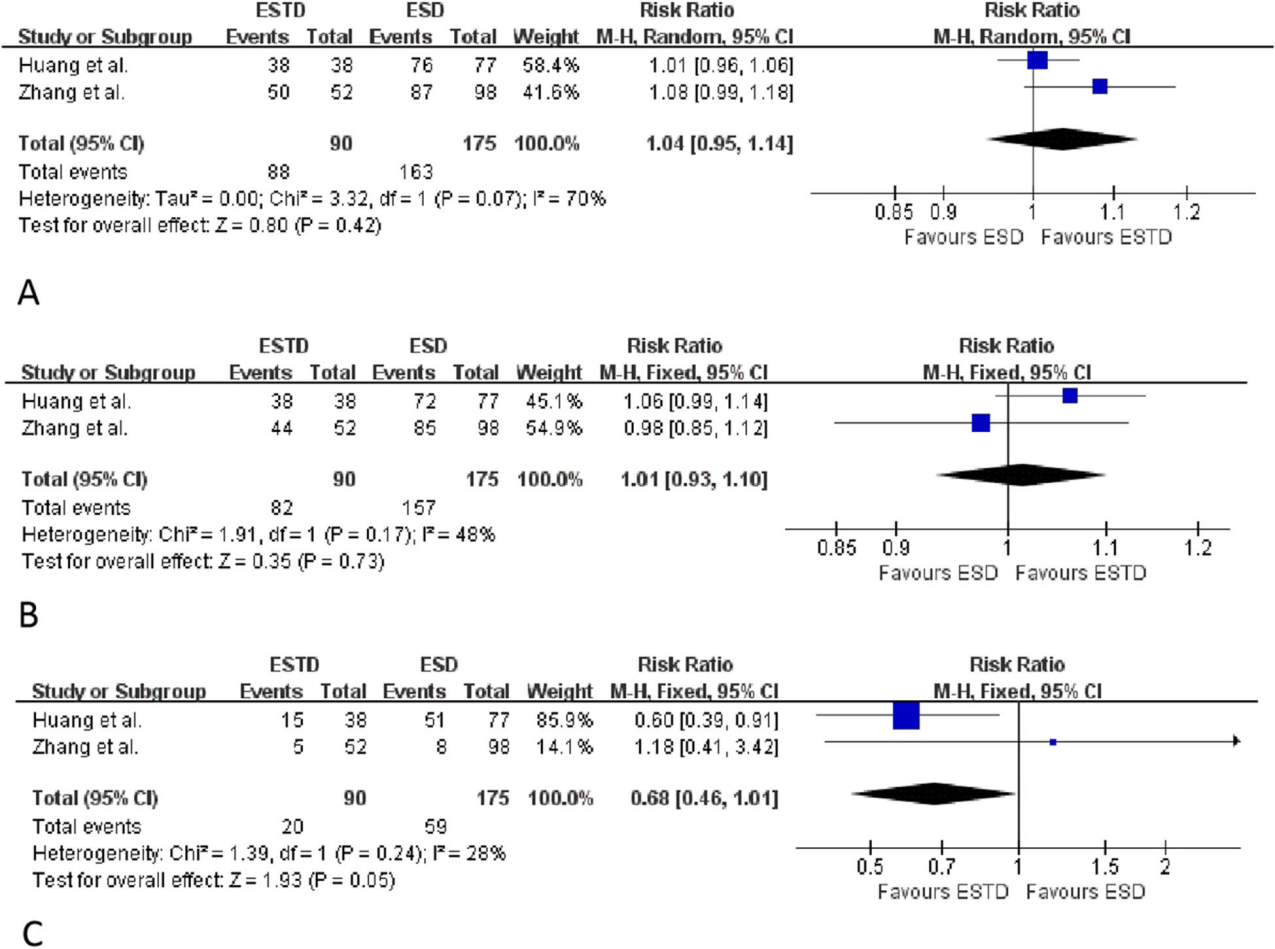
Forest plot to compare en bloc resection rate, R0 resection rate and complication rate between **endoscopic submucosal tunnel dissection** (ESTD) and **endoscopic submucosal dissection (ESD)** groups. **a**, Risk ratios (RR) with 95% CI for en bloc resection was 1.04 (95% CI: 0.95–1.14), Cochran Q test *P* = 0.42, I^2^ = 70%; **b**, For R0 resection, pooled RR was 1.01 (95% CI: 0.93–1.10), Cochran Q test *P* = 0.73, I^2^ = 48%; **c**, As for complications, pooled RR was 0.68 (95% CI: 0.46–1.01), Cochran Q test *P* = 0.05, I^2^ = 28%

A comparison of the efficacy and safety of ESTD vs. ESD for SENLs is shown in Table 5. The RR for en bloc resection was 1.04 (95% CI = 0.95–1.14, Cochran Q test *p* = 0.42, I^2^ = 70%, Fig. 4a). For R0 resection, the pooled RR was 1.01 (95% CI = 0.93–1.10, Cochran Q test *p* = 0.73, I^2^ = 48%, Fig. 4b). For complications, the pooled RR was 0.68 (95% CI = 0.46–1.01, Cochran Q test *p* = 0.05, I^2^ = 28%, Fig. 4c).

**Table 5:** Comparison between endoscopic submucosal tunnel dissection and endoscopic submucosal dissection

Study	Country	Groups	Subjects, n	Mean age, years	Mean operation time, min	Tumor location			En bloc resection, n (%)	R0 resection, n (%)	Complications, n (%)
						Ht	Mt	Lt			
Zhang et al. [12]	China	ESTD	52	61.7	93.21	27	20	5	50 (96.2)	44 (84.6)	Perforation 1, Cardiac mucosal laceration 3, Muscular damage 2, Total 5 (9.6)
		ESD	98	60.59	92.39	41	38	19	87 (88.78%)	85 (86.73%)	Perforation 1, Cardiac mucosal laceration 3, Muscular damage 4, Total 8 (8.2)
Huang et al. [19]	China	ESTD	38	58.7	38.0 (median)	3	22	13	38 (100)	38 (100)	Muscular injury 11, Chest pain 4, Total 15 (39.5)
		ESD	77	59.1	45.0 (median)	12	35	30	76 (98.7%)	72 (93.5%)	Muscular injury 35, Post-procedure bleeding 1, Perforation 4, Chest pain 11, Total 51 (70.8)

*ESTD* endoscopic submucosal tunnel dissection;

*ESD* endoscopic submucosal dissection

*Ht* higher thoracic, *Mt* middle thoracic, *Lt* lower thoracic

### Assessment of study quality

According to the NOS, three studies received a score of 4 points [21–23], two received 5 points [17, 20], one received 7 points [12], and one received 8 points [19].

## Discussion

With the development of the endoscopic technique, ESD has become an optional treatment method for SENLs [25]. But, if the resected mucosa blocks the lumen during surgery, the endoscopic view will become obfuscated, which could increase the difficulty of complete resection [25, 26]. In addition, for lesions that exceed three-fourths of the esophageal wall, the operator must perform multilayer submucosal injections during the procedure, which could prolong the surgical duration and, thus, increase the risk of complications [23]. In order to solve these problems, Von et al. reported the use of ESTD for the treatment of circumferential esophagus lesions in a live porcine model in 2007 [27]. Afterward, Linghu et al. applied this method for the first time in clinical practice in 2013 [28], during which all five patients with SENLs were successfully treated with ESTD with no serious complications or tumor recurrence during a mean follow-up duration of 7.4 months [28]. Since then, more and more clinical research of ESTD has been performed in China [12, 18–20], which might explain why most of the studies included in this meta-analysis were conducted in China. During the ESTD procedure, a dual knife or hybrid knife was used to carefully separate the mucosal layer from the muscularis, thereby creating a submucosal tunnel, through which an endoscope was passed to acquire a clear operative view [27, 28]. Therefore, this novel endoscopic technique can increase the success of en bloc resection, while decreasing the risk of injury to the muscular layer, especially for circumferential SENLs [12, 19].

The results of this meta-analysis confirmed that ESTD is an effective treatment option for SENLs with high en bloc and R0 resection rates. The performance of ESTD was also compared with that of ESD, which showed that the en bloc and R0 resection rates were significantly greater for ESTD. The main difference between the two procedures was the step of lesion resection, although there was no difference in patient status after lesion removal. So, the en bloc and R0 resection rates were similar between ESTD and ESD. However, due to rapid diffusion of the submucosal liquid cushion and an unclear operative view, the ESD procedure may be more time-consuming than ESD [27]. In a study by Huang et al., there was a significant difference in the mean surgical duration between the ESD and ESTD groups (48.0 vs. 38.0 min, respectively, *p* = 0.006) [19].

Due to the narrow lumen, thin wall, and lack of serosa, the esophagus is more vulnerable to perforation than the stomach during an endoscopic procedure [9]. In the present meta-analysis, complications occurred in 279 patients (pooled WPR = 40.0, 95% CI = 25.8–56.1%, *p* < 0.05). The most common complications reported in the studies included muscular injury, ES, and postoperative infection. The incidence of complications was relatively high, but most were managed conservatively. Furthermore, the complication rate was lower with ESTD than ESD. In theory, ESTD can potentially reduce the complication rate due to the following factors [20, 27, 28]. First, the ESTD technique stabilizes the positions of the endoscope and lesion, which can simplify and expedite the procedure. Also, the field of view is clearer with the ESTD technique, which makes it easier to identify small arterioles in the submucosa and prevent bleeding by electric coagulation.

Mizuta et al. reported that ES was the most frequent complication with ESD and the risk of stenosis was significantly higher when the length of the lesion was more than half of the circumference [29]. Subsequently, Ono et al. reported that the risk of ES after circumferential or near-circumferential resection of the esophagus was 88% [30]. In our systematic review, ES was also a common complication with an incidence of 18.8% (95% CI = 15.2–22.9%). Postoperative ES is of most concern for large mucosal defects after ESTD because of reduced quality of life. The size of the lesion and histological depth may be reliable predictors of postoperative stricture [10]. In order to reduce the incidence of postoperative stricture, some scholars have reported different methods for the treatment and prevention of ES after ESD or ESTD, such as oral and local injection/administration of steroids, postoperative placement of retrievable metal stents in the esophagus, transplantation of the oral mucosal epithelium, and injection of mesenchymal stem cells [31]. Abe et al. suggests that oral and local injecting/administration of steroids should be considered as a first-line option for the prevention of ES [32]. Among the included studies, Gan et al. successfully treated ES by placing retrievable metal stents and a water balloon in the esophagus [20]. Ye et al. used esophageal stent placement to prevent post-ESTD esophageal stricture, although postoperative esophageal stricture occurred in 4 (17.4%) patients over a 16-month follow-up period [21]. Therefore, these methods may be sufficient to prevent esophageal stricture after ESTD for circumferential or near-circumferential SENLs.

In addition to esophageal neoplastic lesions, ESTD can also be used to resect submucosal tumors originating from the muscularis propria layer of the upper digestive tract. Many recent studies have reported the efficacy and safety of ESTD for resection of tumors from the upper gastrointestinal submucosal [33–35]. In 2014, Hayashi et al. first reported the use of ESTD for colorectal lateral spreading tumors and named this technique the “pocket-creation method” [36]. In 2017, Bassioukas et al. also reported the successful resection of a large rectal laterally spreading tumor from a 74-year-old woman via endoscopic submucosal dissection (ESD) with a double-tunnel technique [37]. These studies also explored the efficacy and safety of ESTD for the resection of rectal tumors.

There were some limitations to this study that should be addressed. First, most of the included studies were conducted in China, so it may be difficult to apply these findings to Western populations. Also, selection bias could not be excluded. Second, most of the included studies were non-randomized, uncontrolled, and retrospective. Therefore, the overall methodological quality of the published studies was relatively low. Third, the number of subjects who underwent ESTD for SENLs was relatively small (*n* = 494), thus, future studies of larger cohorts are warranted.

## Conclusions

The results of the present study confirmed that ESTD is an efficient therapeutic procedure for SENLs with high en bloc and R0 resection rates. Due to a better view, more efficient vessel coagulation, and more complete resection of the submucosa, ESTD may be safer than ESD for the treatment of SENLs, especially circumferential or near-circumferential lesions. In order to further clarify the superiority of the ESTD technique, more data from randomized controlled trials are needed.

## Data Availability

All data generated or analysed during this study are included in this published article.

## Abbreviations

CI: Confidence intervals
EMR: Endoscopic mucosal resection
ES: Esophageal stricture
ESD: Endoscopic submucosal dissection
ESTD: Endoscopic submucosal tunnel dissection
NOS: Newcastle-Ottawa Scale
PRISMA: systematic reviews and meta-analyses
RR: Risk ratios
SENL: Superficial esophageal neoplastic lesions
WPR: Weighted pooled rates
